# One-step reverse transcription loop-mediated isothermal amplification (RT-LAMP) for detection of tomato torrado virus

**DOI:** 10.1007/s00705-016-2774-2

**Published:** 2016-02-18

**Authors:** Marta Budziszewska, Przemysław Wieczorek, Aleksandra Obrępalska-Stęplowska

**Affiliations:** Interdepartmental Laboratory of Molecular Biology, Institute of Plant Protection-National Research Institute, Władysława Wegorka 20, 60-318 Poznan, Poland

**Keywords:** Torradovirus, RT-LAMP, Plant virus detection, Virus diagnostics, Diagnostic protocol

## Abstract

‘Torrado’ disease caused by tomato torrado virus (ToTV) is responsible for considerable losses in tomato production. Therefore, a one-step reverse transcription loop-mediated isothermal amplification protocol for early and fast detection of ToTV isolates has been developed. The RNA extracted from ToTV-infected plants was tested using this protocol with a set of six primers specific for the Vp35 coat protein gene sequence. The amplified products were analyzed using amplification curves, electrophoresis, and direct staining of DNA. The sensitivity of the protocol was tenfold higher than that of conventional RT-PCR. This new protocol is inexpensive, rapid, simple, and very sensitive.

## Introduction

Tomato torrado virus (ToTV) belongs to the genus *Torradovirus* in the family *Secoviridae* [1]. It is considered a major tomato pathogen worldwide. Symptoms of ToTV infection in tomato begin with the yellowing of the leaflet base, which develops into necrosis of the whole plant, including fruits, often causing its death [1, 2]. The ToTV genome consists of two positive single-stranded RNA sequences, which are designated RNA1 and RNA2. Both RNA sequences are polyadenylated at the 3′ end. RNA1 contains a single open reading frame (ORF1) that encodes a polyprotein with domains for viral replication proteins. RNA2 consists of two ORFs, with the first encoding a protein of unknown function and the second encoding a polyprotein with domains for a movement protein and three coat protein subunits, namely, Vp23, Vp26, and Vp35 [1, 2].

Tomato torrado virus has been reported in France [3], Hungary [4], Poland [5], Spain [6–8], Italy [9], Australia [10], Colombia [11], Panama [12], and South Africa [13]. However, based on available ToTV sequence data, the majority of identified isolates originated from Spain, where the virus is widely distributed. Because of its wide distribution and virulence, ToTV control strategies have focused on the development of effective diagnostic methods aimed at early detection. Previously described ToTV diagnostic protocols are based on double-antibody sandwich enzyme-linked immunosorbent assays [14], reverse transcription polymerase chain reactions (RT-PCR) [1, 8, 11, 15], immunocapture [5, 16], molecular hybridization with dig-RNA probes [8], and real-time PCR-based methods involving high-resolution melting analysis [17] and TaqMan probes [18].

An interesting alternative to PCR-based technologies is loop-mediated isothermal amplification (LAMP), which is a molecular technique developed in 2000 [19]. This method involves a one-step amplification of target DNA completed under isothermal conditions. It is a highly efficient and fast protocol that is specific for the target sequence because of the use of four or six primers targeting six or eight different regions, respectively [19, 20]. Moreover, if used with thermostable reverse transcriptase, this method can be applied to detect pathogens with an RNA genome [19, 21]. To date, RT-LAMP has been used to detect several plant viruses, including members of the genera *Potyvirus* [21, 22], *Comovirus* [23], *Ilarvirus* [24], and *Crinivirus* [25].

In this study, we developed molecular tools based on RT-LAMP for rapid and specific ToTV detection. We also compared the sensitivities of RT-LAMP and the standard RT-PCR approach.

The nucleotide sequences of known torradoviruses were retrieved from GenBank and aligned to identify conserved ToTV genomic regions. A set of ToTV-specific primers was designed using LAMP Designer 1.12 software. The *Vp35* gene was chosen as the amplification target. The RT-LAMP assay was carried out using the following primers: forward outer F3_Vp35 (5′-ACCAACCCATATCCTCCC-3′), reverse outer B3_Vp35 (5′-CCTTACAGCTTCATTGGCA-3′), forward inner FIP_Vp35 (5′-GCCTGCTCCTTTGCCACATTGATTGTTATGATGGCTTAACG-3′), reverse inner BIP_Vp35 (5′-GTGGCCCAAACTAGTGTGGAATTCATGCTATCCACACTGC-3′), loop forward LoopF_Vp35 (5′-CTCTAGCTCACTGCGAACTT-3′), and loop reverse LoopB_Vp35 5′-ATACCATCCACCTCATTCGC-3′. The ToTV isolates used for the amplifications were as follows: three Polish isolates, Wal′03 (EU563947) [2], Kra (KJ940974), and Ros (KM114266) [26]); four Spanish isolates, ALM04 (GQ397437), ALC07 (GQ397442), MUR07 (GQ397443), and MUR05 (GQ397439) [8]; four recombinant infectious clones based on the Kra2014 ToTV RNA2 sequence [27]; the mutants ToTV-Kra sec1 and sec3 (the Vp35 amino acid sequences were derived from ToTV isolates sec1 [KJ571198] and sec3 [KJ571200], respectively); and ToTV-Kra-G759A (generated in our laboratory). Tomato marchitez virus (ToMarV) [28] and tomato apex necrosis virus (ToANV; EF063642) [29], which is believed to be a ToMarV strain, were used as negative controls. ToMarV has been isolated from tomatoes in Mexico and is closely related to ToTV according to phylogenetic analysis. Additionally, the total RNA isolated from carrot infected with carrot torradovirus (CaTV) [30], a non-tomato-infecting (NTI) torradovirus was also used as a negative control.

**Fig. 1:**
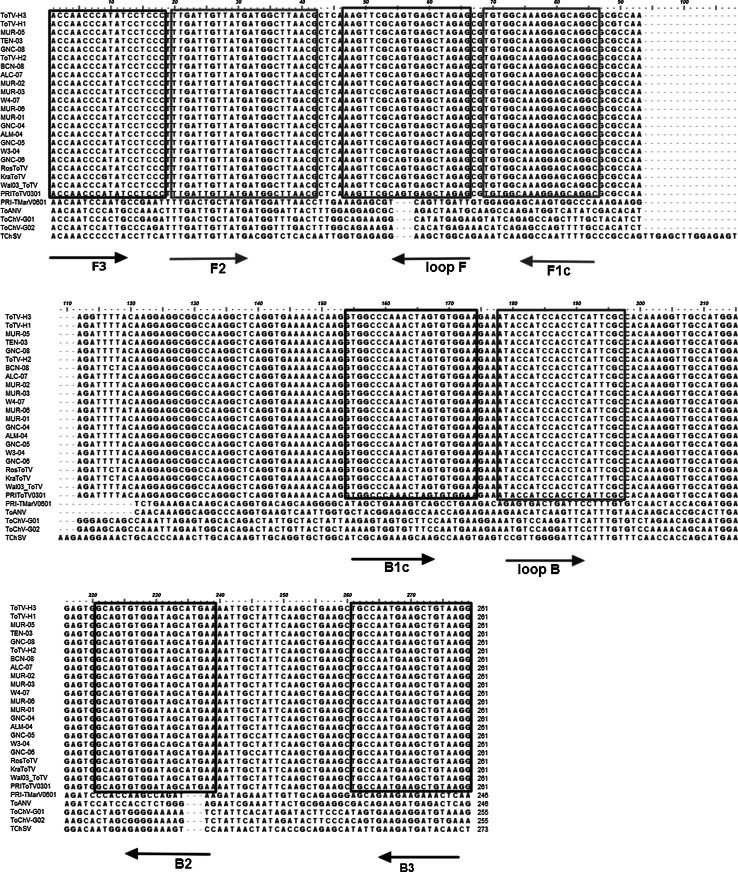
Multiple sequence alignment of the Vp35 coat protein subunits of 22 isolates of tomato torrado virus and other torradoviruses. The RT-LAMP primers used in this work were designed based on conserved genome regions (boxed)

Multiple sequence alignments using available torradovirus sequences revealed considerable genetic differences between ToTV and the other torradoviruses within the target region, which confirmed that the primers were ToTV-specific (Fig. 1). An *in silico* BLASTN analysis [31] of the LAMP primers also confirmed a lack of homology with sequences from other members of the genus *Torradovirus*. Total RNA from infected and healthy tomato tissues was isolated using TRI Reagent (ThermoFisher Scientific, Waltham, Massachusetts, USA) according to the manufacturer’s instructions. The RNA quality and quantity were assessed using a NanoDrop spectrophotometer (ThermoFisher Scientific, Waltham, Massachusetts, USA), and the final concentration was adjusted to approximately 150 ng/µl. We performed the RT-LAMP assay in a single tube containing a total volume of 25 µl. The reaction mixture consisted of 2 μl of 10 µM FIP_Vp35 and BIP_Vp35, 0.5 μl of 10 µM F3_Vp35 and B3_Vp35, and 1 μl 10 µM LoopF_Vp35 and LoopB_Vp35 primers; 15 µl of Isothermal MasterMix (ISO-001), containing Gsp SSD polymerase and thermostable pyrophosphatase, fluorescent dye (OptiGene, Horsham, UK), 1 μl of template, 0.25 µl (1 U/µl) of LAMP reverse transcriptase (appropriate for RT-LAMP, optimal temperature: 63 °C) (Novazym, Poznan, Poland); and water. The tube was incubated at 63 °C for 30 min using a water bath or a Bio-Rad CFX96 Touch^TM^ Real-Time PCR Detection System (Bio-Rad, Hercules, California, USA). The thermal profile of the reaction done in real-time machine consisted of a preincubation step at 63 °C for 60 s, followed by 50 cycles of 30 s at 63 °C.

To assess the sensitivity of the RT-LAMP assay, the concentration of total RNA from tomato plants infected with Kra-ToTV was adjusted to 100 ng/μl, and this preparation was serially diluted in a solution of total RNA isolated from a healthy plant (100 ng/μl). The RT-LAMP protocol was completed as described using 1 µl of serially diluted templates. Additionally, a conventional one-step RT-PCR was performed using a Transcriptor One-Step RT-PCR Kit (Roche Diagnostics, Poland) and ToTV-specific 2TT5/6 primers previously designed by Budziszewska et al. [2]. The reactions were carried out in a total volume of 25 μl with 0.4 μM primers, 1× RT-PCR reaction buffer, and 1 μl of Transcriptor enzyme mix. The RT-PCR program consisted of a reverse transcription step at 50 °C for 30 min, followed by denaturation at 94 °C for 7 min; 10 cycles of 94 °C for 10 s, 55 °C for 30 s, and 68 °C for 40 s; and 25 cycles of 94 °C for 10 s, 55 °C for 30 s, and 68 °C for 60 s. The program was completed with an extension step at 68 °C for 10 min. The amplified products obtained from the RT-LAMP assay and the RT-PCR reaction were analyzed by electrophoresis in a 1.5 % agarose gel and were visualized using Midori Green (Nippon Genetics GmbH) under UV light. We also visually assessed the amplification products of the RT-LAMP assay using 0.5 μl of the final LAMP product and 20 μl of 1× EvaGreen staining solution (Biotium). The samples were observed using a UV lamp.

**Fig. 2:**
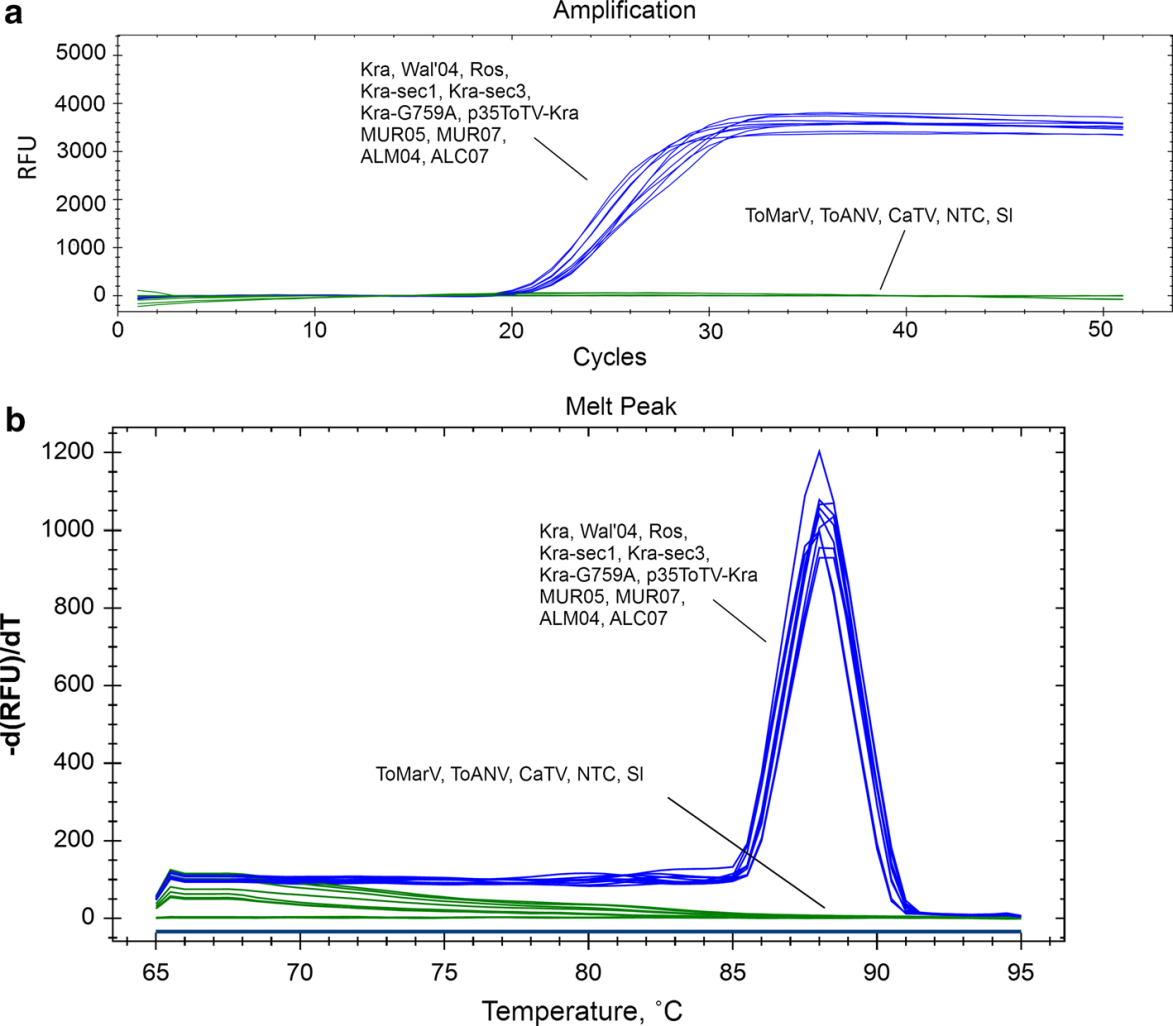
Real-time monitoring of the RT-LAMP assay results. Amplification plots and dissociation curves of RT-LAMP products are shown in panels A and B, respectively. The ToTV isolates used as positive controls (blue curves) included Spanish isolates (MUR05, MUR07, ALC07, and ALM04), Polish isolates (Kra, Ros, and Wal′03), and infectious clones based on RNA2 of Kra-ToTV (p35ToTV-Kra, Kra-sec1, Kra-sec3, and Kra-G759A). The negative controls (green lines) consisted of tomato apex necrosis virus (ToANV), tomato marchitez virus (ToMarV), carrot torradovirus (CaTV), a healthy tomato plant (Sl), and a no-template control (NTC)

**Fig. 3:**
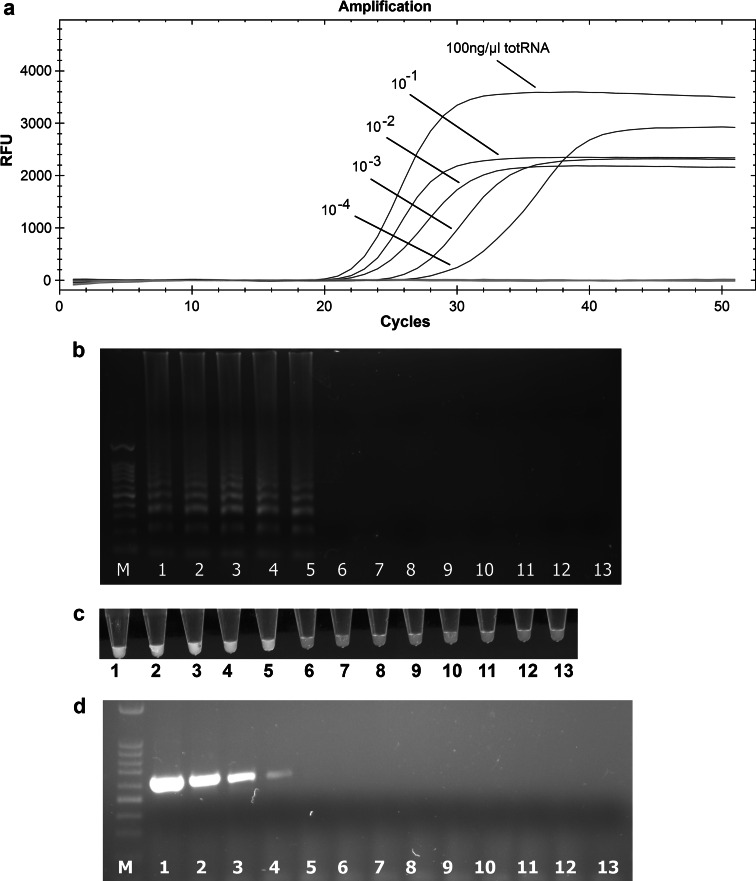
Sensitivity comparison between the RT-LAMP assay and the RT-PCR method. The detection limit of the RT-LAMP assay was determined based on real-time amplification plots (a), electrophoresis (b), DNA staining followed by visual assessment (c), and one-step RT-PCR (d). M, Nova 100-bp molecular weight marker (Novazym, Poland); lane 1, total RNA 100 ng/µl; lanes 2–11, tenfold serial dilutions of total RNA; lanes 12-13, negative samples (healthy plant, no template control). The order of samples in panel d is the same as in panel b

The LAMP assay has been used for the molecular detection and diagnosis of many pathogens, including bacteria, viruses, fungi, and parasites responsible for plant, animal, and human diseases [20, 32, 33]. Our results revealed that the target Vp35 gene of ToTV isolates can be rapidly amplified in 12-25 minutes, depending on the virus concentration (Fig. 3). The amplification plots of positive samples were observed between the 20th and 22nd cycle, and no significant difference between tested ToTV isolates was observed. The negative controls gave no positive signals (Fig. 2). No amplification products were observed for RNA isolated from ToANV-, ToMarV-, and CaTV-infected plants, total RNA isolated from healthy plants, or no-template controls (Figs. 2 and 3). The new RT-LAMP assay is 10 times more sensitive than RT-PCR. The RT-LAMP allows detection of ToTV in as little as a 10^-4^ dilution of total RNA, whereas the detection limit of conventional one-step RT-PCR was about 10^-3^ (Fig. 2). This corresponds well to the RT-LAMP sensitivity reported for other plant viruses (21, 23, 24). Recent studies by Herrera Vásquez et al. showed that the ToTV detection limit of real-time PCR detection with TaqMan probes ranged from 10^3^ to 10^10^ ToTV RNA copies [18]. These data suggest that the detection limit of these methods might be comparable. An important feature of the RT-LAMP method is the very short detection time. Despite the fact that ToTV RT-LAMP detection requires prior RNA isolation from tested plant samples, which makes it impossible to perform under field conditions, this technique is still less time-consuming than standard RT-PCR or even real-time RT-PCR. Furthermore, RT-LAMP does not require expensive equipment (e.g., a real-time PCR machine or a thermal cycler), and it may be performed successfully using a water bath or thermoblock.

In conclusion, the RT-LAMP assay developed in this study is a rapid, cost-effective, efficient, and simple method to detect ToTV and may be a useful tool for monitoring of ‘torrado’ disease. Moreover, this is the first report describing the use of an RT-LAMP assay to detect ToTV in infected tomato plants.
